# Relationship Between Poor Visual Acuity and Lifestyle: A Longitudinal and Cross-Sectional Study of Japanese Students Using Secondary Data

**DOI:** 10.7759/cureus.79980

**Published:** 2025-03-03

**Authors:** Misaki Shiraishi, Hiromi Kawasaki, Sae Nakaoka, Satoko Yamasaki, Nanami Funaki, Iori Masukane

**Affiliations:** 1 Department of Health Science, Graduate School of Biomedical and Health Sciences, Hiroshima University, Hiroshima, JPN

**Keywords:** japanese student, lifestyle, myopia, poor vision, poor visual acuity

## Abstract

Background

The incidence of myopia-related vision loss among Japanese children is increasing, indicating that efforts to prevent this vision loss remain insufficient. School-aged Japanese children are thought to be more prone to myopia due to physical and lifestyle changes during their growth stages. This study aimed to clarify the relationship between changes in visual acuity (VA) from fourth-grade elementary school to junior high school and students' lifestyle habits and to examine efforts to maintain visual acuity among elementary and junior high school students in Japan.

Methods

The participants of the analysis included 49 Japanese junior high school students. Secondary data used in the study were the results of vision tests and lifestyle questionnaires administered at the students' elementary and junior high schools. A longitudinal analysis was performed by comparing visual acuity and lifestyle conditions at two time points: fourth grade of elementary school and junior high school. Furthermore, the relationship between changes in visual acuity from elementary to junior high school and lifestyle habits in junior high school was examined.

Results

Poor visual acuity in junior high school was significantly associated with poor visual acuity in the fourth grade (p=0.003). Students with poor vision (PV) had a significantly higher percentage of the following characteristics compared to those without poor vision: studying for more than two hours per day on their days off (p=0.043), spending less than one hour per day on the computer on weekdays (p=0.020), perceiving insufficient sleep duration (p=0.018), and not paying attention to taking breaks while studying or watching TV (p=0.038).

Conclusion

This study indicated that poor vision in school-aged children may progressively worsen without improvement. The poor vision group spent more time studying on weekends and less time on the computer during the week, reported insufficient sleep time, and neglected taking breaks while studying or watching TV. As students grow, they tend to spend more time studying and changing their living environment to one that demands the overuse of their eyes, increasing strain on them. It is necessary to teach students to prevent poor vision starting at school age while their eyesight is still good.

## Introduction

The global prevalence of myopia is steadily increasing and is expected to reach 4.758 billion (49.8% of the world's population) by 2050, of which 938 million (9.8% of the world's population) are expected to develop high myopia [1], highlighting it as a global health problem. The younger the age at myopia onset, the greater the risk of developing high myopia. All children who develop myopia before the age of 10 years are at risk of high myopia and require attention [2]. High myopia is associated with a risk of complications, such as myopic maculopathy, glaucoma, myopia-related glaucoma-like optic neuropathy, and retinal detachment. Myopia and myopia-related complications reduce the quality of life [3]. Particularly in children whose myopia remains inadequately corrected, it can influence school performance, limit future employability, and impair quality of life [4]. Myopia affects both children's present lives and future prospects.

The onset and progression of myopia result from complex interactions between genetic and environmental factors [5]. Genetic factors include ethnicity, sex, and family history of myopia [6]. Environmental factors include living environment, educational level, socioeconomic status, near-vision work, and outdoor activities [5]. While genetic factors are beyond our control, environmental factors can be altered through the awareness and support of one's surroundings; therefore, preventive interventions are necessary.

The shape of the eye lens adjusts when focusing on an object. Frequent changes in the shape of the lens generate sustained pressure on the ocular wall, which causes axial elongation [7]. Myopia develops when the ocular axial length increases beyond the normal length. Overextended ocular axial length is irreversible. As school-aged children age, they engage in more activities requiring them to focus on close objects, which strains their eyes. The length of the ocular axis develops soon after birth and stabilizes around the age of 10 [8]. The majority of myopia cases observed in school-aged children are ocular axial myopia [8], and the risk of myopia may increase due to excessive near-viewing during ocular axis development. An increase in the height and eye axis length positively correlates with the transition from childhood to adolescence [9]. With age, the bodies of children develop and grow. Particularly during school age, children's height increases significantly, leading to changes in axial length, which may make them more susceptible to myopia.

In Japan, October 10 is observed as "Eye Protection Day" every year, and the national government and the Japanese Ophthalmologists' Association lead educational activities related to eye health [10] and provide guidance at home and schools, through ophthalmologists. The percentage of children with a visual acuity (VA) of <1.0 has been consistently increasing since 1979. In that year, 17.91% of elementary school students, 35.19% of middle school students, and 53.02% of high school students had visual acuity below 1.0 [11]. By 2022, these percentages had risen to 37.88% for elementary school students, 61.23% for middle school students, and 71.56% for high school students [12]. The 2022 figures represent the highest percentages ever recorded for each educational level. Myopia is the most common cause of visual acuity in children [13]. A study of 726 elementary school students and 752 junior high school students in Tokyo, Japan, reported that the prevalence of myopia in 2017 was 76.5% among elementary school students and 94.9% among junior high school students, which is interpreted as rates higher than ever before [13]. The incidence of myopia-related vision loss among Japanese children is increasing, indicating that the situation does not protect their eyesight. Even with such teaching, the prevalence of myopia among Japanese children has not decreased. It is therefore necessary to assess whether the instructions are being followed by the children and align with their actual living conditions.

Children today have been surrounded by digital technology from birth, and their daily lives and habits are intertwined with the use of social media, smartphones, tablets, and the Internet [14]. In 2019 the Japanese government initiated efforts to promote the informatization of education, including the introduction of digital textbooks and the creation of a digital terminal environment for each student. The impact of increased digital device screen time has been identified as an environmental factor affecting myopia [15]. It can be inferred that Japanese children growing up with more digital devices around them are more susceptible to myopia. Moreover, the use of digital technology is expected to increase in the future. We believe that the same is true for children's learning and daily lives. The risk of myopia in children living in digitalized societies is estimated to be higher than before. It is estimated that school-aged Japanese children are more prone to myopia because of physical and lifestyle changes associated with growth. In particular, height growth peaks in the upper elementary and junior high school years, and the length of the ocular axis tends to change. From elementary to junior high school, the amount of learning and class time increases, along with an increase in activities that strain the eyes. The stress on the eyes is further increased by digitalized lifestyles. Therefore, the approach to preventing vision loss should account for changes over time in children from elementary school through middle school.

This study compared visual acuity and lifestyle habits of junior high school students to those they had in the fourth grade. This study clarified the relationship between visual acuity and lifestyle and examined efforts to maintain visual acuity among elementary and junior high school students in Japan.

## Materials and methods

Design

This is a longitudinal and cross-sectional study. By comparing the visual acuity and living conditions of the same students in fourth grade and junior high school, our study examined longitudinal changes at two points in time. The cross-sectional design of the study examined the relationship between changes in visual acuity from fourth-grade elementary school to junior high school and lifestyle in junior high school.

Sample

The study population consisted of 62 fourth-grade students enrolled in an elementary school A, in April 2019; those who did not participate in the vision screening conducted by the school were excluded. Additionally, to ensure data linkage to their middle school period, this study excluded individuals who did not enroll in junior high school B in 2023, four years later, and those who did not participate in the vision screening conducted by the school.

Data collection

This study used secondary results from a survey conducted in elementary school A and junior high school B in Japan. This study used two surveys: a visual acuity (VA) test and a self-administered questionnaire on actual living conditions (lifestyle survey). These two surveys were conducted as part of the school education program. For the purpose of student healthcare, teachers monitored each student’s condition individually. After concatenating the results for the same students, the school nurse-teacher prepared a database with anonymous numbers and provided it to the researchers. Participants' VA data were collected from their regular school physical examination records. Visual acuity was measured at 5 m using the Landolt C-chart. VA tests were conducted in April 2019 and 2023. The lifestyle survey was conducted in elementary school A in February 2020, and the same survey was conducted in junior high school B in November 2022. These surveys were conducted by elementary school A and junior high school B, targeting all fourth-grade and junior high school students enrolled at the time.

**Figure 1:**
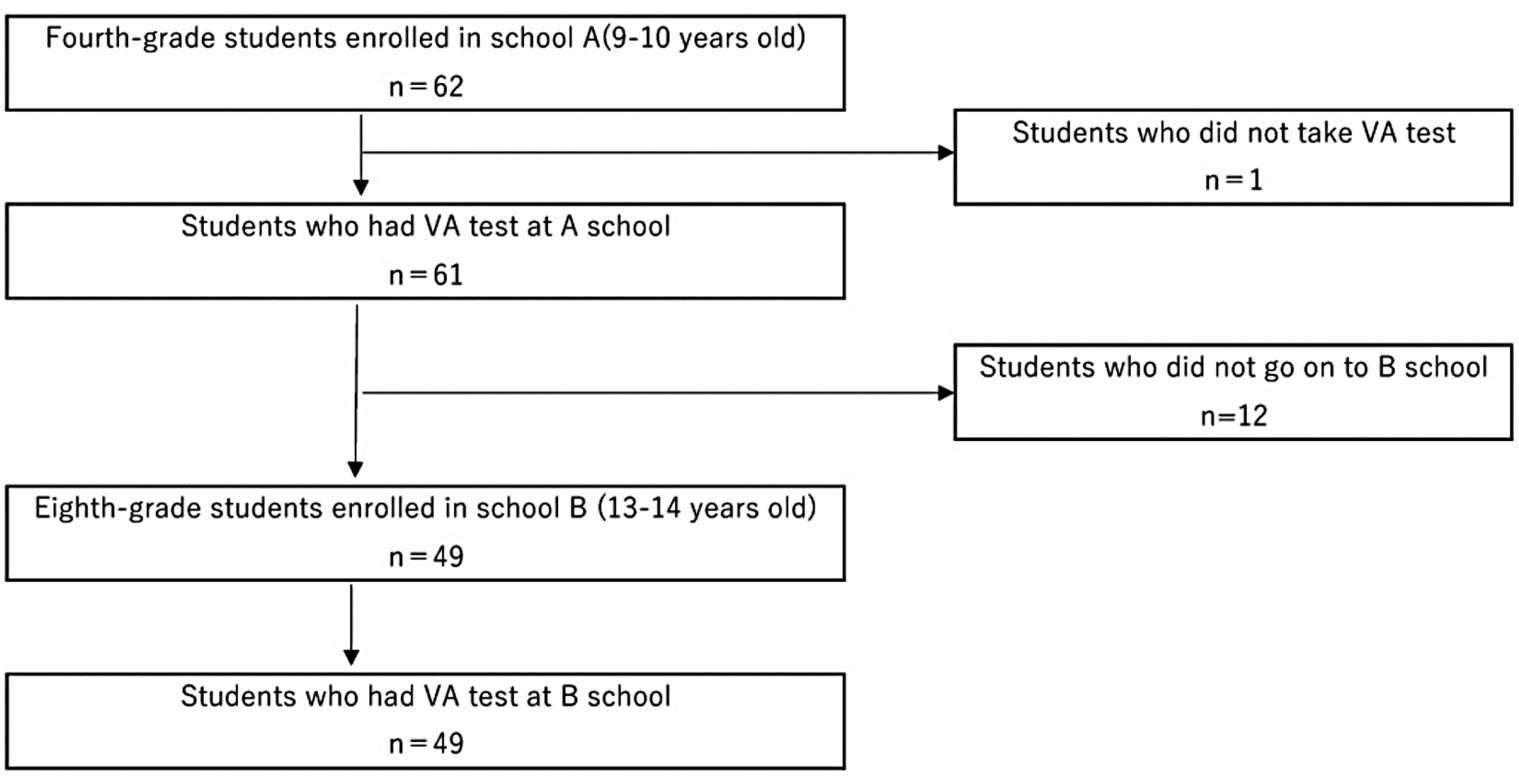
Flow diagram showing the number of participants analyzed VA: visual acuity

The analysis was conducted on 49 participants for whom the results of vision tests in fourth-grade elementary school and junior high school were available for linkage (Figure 1). The lifestyle survey was answered by all participants (49/49 (100%)) in fourth grade and by 32/49 (65.3%) in junior high school. Consequently, 32 participants were included in the analysis to examine the relationship between visual acuity and living conditions. In cases of missing responses, they were excluded from the analysis for that question item only.

Contents of data used

Secondary data are presented in Table 1, followed by the details of the data.

**Table 1:** List of data used VA: visual acuity

Implementation year (sample)	Usage data
2019 (fourth-grade students)	VA test, lifestyle survey
2022 (middle school students)	Lifestyle survey
2023 (middle school students)	VA test

VA Test

Visual acuity was scored on a five-point scale: A (visual acuity ≥ 1.0), B (0.7 to 0.9), C (0.3 to 0.6), D (＜0.3), and those who had corrected their vision with glasses or other means. Humans typically have a dominant eye, and studies indicate it is usually the right eye [16,17]. Therefore, this study used the visual acuity data of the right eye.

Lifestyle Survey

This survey was completed by the schools. The survey included basic attributes (grade, sex, and parents' vision correction status) and life situations over the past month.

Life situations consisted of six items: (1) study and reading habits, (2) digital device usage, (3) indoor and outdoor activity habits, (4) sleep status, (5) stress response, and (6) awareness of behaviors to prevent poor vision (PV).

The students rated the number of books read and time spent reading and studying on a four-point scale, with scores ranging from one to four. Higher scores indicate more time spent reading and studying.

Moreover, they rated time spent using TV/video, game consoles (mobile phones), and PCs (Internet games and tablet PCs) on a five-point scale, with scores ranging from one to five. Higher scores indicate more time spent using digital devices.

Furthermore, students were asked to rate their exercise and sports activities, excluding school lessons, and the time spent playing outside on a four-point scale, with scores ranging from one to four. The number of days of outdoor play was rated on a five-point scale, with scores ranging from one to five. Higher scores indicate more time and frequency of exercise and outdoor play.

Students' sleep status was assessed using the Japanese version of the Athens Insomnia Scale [18]. It consists of eight items that measure sleep conditions over the past month, including ease of falling asleep, daytime sleepiness, and nocturnal awakening. Students responded on a four-point scale, with scores ranging from one to four. The higher the score, the more likely the student had insomnia, impaired nighttime sleep, or impaired daytime functioning.

The Stress Response Scale for Elementary School Students developed by Shimada et al. [19] was used to ascertain the stress reactions. It consists of items measuring "physical reactions" (five items), "depressed and anxious feelings" (five items), "bad mood and angry feelings" (five items), and "apathy" (five items). Respondents were asked to rate their mental and physical health over the past month on a four-point scale ("Not at all," "Almost never," "Sometimes," and "Often"). The higher the score, the more stress reactions the student expressed.

The students were also asked about their awareness of behaviors that are considered necessary to prevent vision loss. This included a total of eight questions. These were rated on a four-point scale ("Not at all concerned," "Not very concerned," "Sometimes concerned," and "Often concerned") and scored on a scale of 0 to three. Higher scores indicate higher student awareness of actions to prevent poor vision.

Statistical analysis

Analysis Method by Content

Visual acuity at fourth grade in terms of junior high school students' visual acuity: This study examined whether junior high school students with poor vision had poor vision in the fourth grade. As shown in Table 2, the middle school students were divided into two groups: those whose test results were A, classified as "Good Visual Acuity (GVA)" and those whose test results were B, C, or D and those with corrected vision, classified together as "Poor Visual Acuity (PVA)."

**Table 2:** Visual acuity classification GVA: Good Visual Acuity, PVA: Poor Visual Acuity

Visual acuity classification		Group
A	Unaided visual acuity is classified to be 1.0 or more in both eyes.	GVA
B	Unaided visual acuity is classified to be 0.9 to 0.7.	PVA
C	Unaided visual acuity is classified to be 0.6 to 0.3.	
D	Unaided visual acuity is classified to be 0.2 or less.	
	Those who use eyeglasses or contact lenses.	

Differences between the lifestyle of junior high school students and the lifestyle they had when they were in the fourth grade: This study considered if there exists any difference between the lives of middle school students and their lives when they were in fourth grade.

Each item in the lifestyle survey was scored, and the total score was calculated for each item. The total scores of middle school students and fourth graders were compared. After confirming normality with the Shapiro-Wilk test, a corresponding t-test was used for items with normal distribution and Wilcoxon's signed-rank test for the other items.

Relationship between the changes in junior high school students' visual acuity since fourth grade and the lifestyle of junior high school students: The middle school students were divided into two groups. The visual acuity test results from junior high school students were consolidated with their fourth-grade results. Next, the visual acuity of the junior high school students was assessed (Table 3), designated as point A. Similarly, the visual acuity of the fourth-grade students was scored (Table 3), marked as point B. Score A was subtracted from score B. Consequently, the entire sample was divided into two groups, as shown in Table 4. Comparisons were made between the two groups, "not poor vision (NPV)" and "poor vision (PV)" for each of the basic attributes and middle school student lifestyle survey. Unanswered questions were excluded from analysis. Either Kruskal-Wallis or χ^2^ tests were used for testing.

**Table 3:** Visual acuity classification and scores

Visual acuity classification		Points
A	Unaided visual acuity is classified to be 1.0 or more in both eyes.	1
B	Unaided visual acuity is classified to be 0.9 to 0.7.	2
C	Unaided visual acuity is classified to be 0.6 to 0.3.	3
D	Unaided visual acuity is classified to be 0.2 or less.	4
	Those who use eyeglasses or contact lenses.	5

**Table 4:** Grouping of subjects Score A: visual acuity points at junior high school age, score B: visual acuity points at fourth grade, NVP: not poor vision, VP: poor vision

Score B minus Score A	Group
0 to 4 points	NPV
-1 to -4 points	PV

Statistical analyses were performed using IBM SPSS Statistics for Windows version 27.0 (IBM Corp., Armonk, NY). Statistical significance was set at p<0.05.

Ethical consideration

This study was conducted in accordance with the guidelines of the Declaration of Helsinki and approved by the Hiroshima University Epidemiological Research Ethics Review Committee (approval number: E2018-1251-2). This study made secondary use of longitudinal data previously collected by the school for managing student health. The data were analyzed following approval for secondary use from the school principal. Students and their parents are informed by the school principal about the university's secondary use of these data. This represents an effective approach to utilizing existing data accumulated in schools.

## Results

VA at fourth grade in terms of visual acuity in junior high school

No significant gender differences in GVA and PVA were observed in fourth-grade students (p=0.680). Similar results were found in junior high school students (p=0.098). Consequently, boys and girls were analyzed together in this study.

Students with poor VA in junior high school had already exhibited poor VA in fourth grade (p=0.003) (Table 5).

**Table 5:** Comparison of visual acuity between middle school and fourth grade Analysis: chi-squared test *p<0.05 (significant) GVA: Good Visual Acuity, PVA: Poor Visual Acuity

		Fourth-grade students				p-value
		GVA		PVA		
		Number	%	Number	%	
Middle school students	GVA	17	73.9	6	26.1	0.003*
	PVA	8	30.8	18	69.2	

Differences between the lifestyle of junior high school students and their fourth-grade self

**Figure 2:**
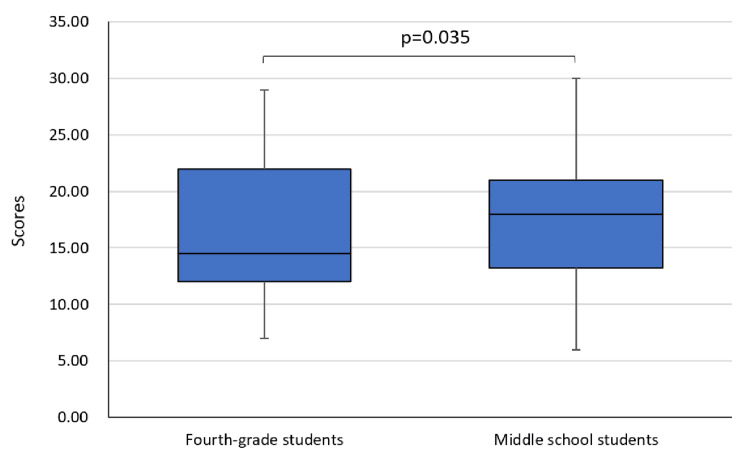
Comparison of digital device scores between middle school and fourth grade Analysis: Wilcoxon's signed-rank test

As shown in Figure 2, middle school students' digital device scores were significantly higher than their scores in fourth grade (p=0.035). Time spent using information devices significantly increased during junior high school compared to fourth grade.

**Figure 3:**
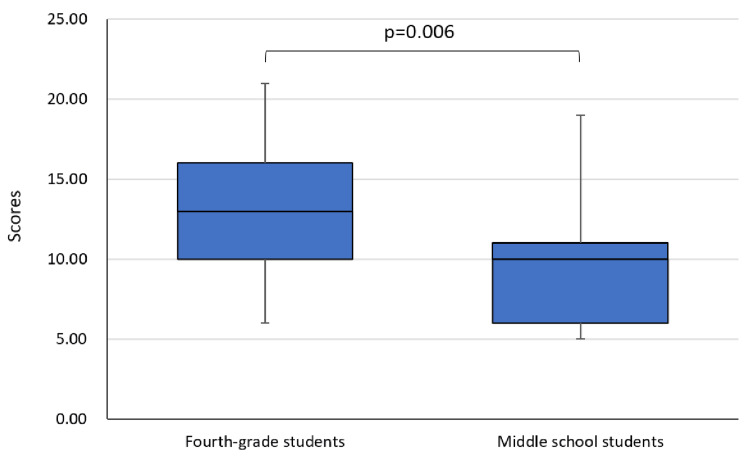
Comparison of physical activity scores between middle school and fourth grade Analysis: Wilcoxon's signed-rank test

Moreover, the physical activity scores of the middle school students were significantly lower than those in fourth grade (p=0.006) (Figure 3). During junior high school, the time and frequency of indoor and outdoor activities were significantly lower than those in fourth grade.

**Figure 4:**
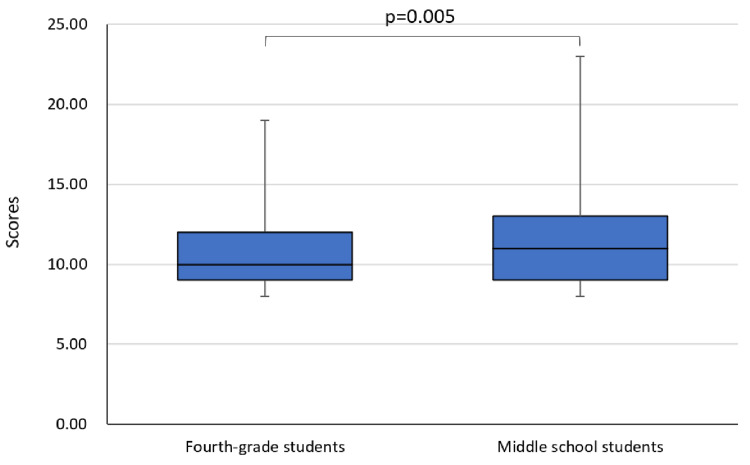
Comparison of sleep scores between middle school and fourth grade Analysis: Wilcoxon's signed-rank test

As shown in Figure 4, significant differences were found between sleep scores in junior high school and fourth grade (p=0.005). Middle school students experienced significantly more sleep difficulties than fourth-grade students.

No significant difference was noted between the scores for practices to prevent vision loss in junior high school and fourth grade (p=0.237). Middle school students did not differ in their awareness regarding behaviors to prevent vision loss, as they did in fourth grade.

Relationship between lifestyle in junior high school and changes in visual acuity since fourth grade

Given that there was no significant difference between NPV and PV by sex (p=0.299), this study examined boys and girls in combination.

As shown in Table 6, poor vision (PV) had a significantly higher percentage of "those who study more than two hours/day on their days off" (p=0.043), "those who spent less than one hour/day on the computer on weekdays" (p=0.020), "those who felt they did not get enough sleep length" (p=0.018), "those who do not pay attention to breaks while studying or watching TV" (p=0.038) than NPV. Both PV and NPV did not differ significantly according to parental vision correction status (p=0.939).

**Table 6:** Comparison of NPV and PV lifestyle when they were in middle school Analysis: chi-squared test *p<0.05 (significant) NPV: not poor vision, PV: poor vision

Life of junior high school students		NPV		PV		p-value
		Number	%	Number	%	
Study time on holidays	<2 hours/day	17	89.5	7	58.3	0.043*
	≤2 hours/day	2	10.5	5	41.7	
Computer usage time on weekdays	<1 hour/day	5	25.0	8	66.7	0.020*
	≤1 hour/day	15	75.0	4	33.3	
Sleeping time	Enough	9	50.0	1	8.3	0.018*
	Shortage	9	50.0	11	91.7	
Break during near-sighting work	Not conscious	7	36.8	9	75.0	0.038*
	Consciousness	12	63.2	3	25.0	

## Discussion

This study examined the relationship between changes in visual acuity and the lifestyle status of elementary and junior high school students in Japan. These results indicate that poor vision in school-aged children may progressively worsen without improvement. This study discusses the measures required to prevent vision loss among elementary and junior high school students in Japan.

Individuals with reduced vision at school age may continue to experience reduced vision in subsequent years. Interventions to prevent myopia onset should be implemented during elementary school, especially by third grade [20]. Moreover, individuals with reduced vision during school-age years should be considered at high risk for vision loss.

PV was not affected by the parental myopia status, which contradicts the findings of previous studies [21]. Parental habits and lifestyles notably affect children. Children of myopic may grow in an environment that is conducive to myopia [22], suggesting that vision loss may be related to environmental factors rather than genetic factors. The results of this study revealed several environmental factors. The first environmental factor influencing poor vision is study habits. The PV group had longer study time on holidays. As the students advanced from elementary to junior high school, the duration spent on studies also increased, and it is possible that near-vision work time increased with extended study hours, resulting in visual acuity loss. It is necessary to teach children time management skills to reduce the duration of near-vision work in less time. The next factor is the use of digital devices. Students spend more time using digital devices as they advance from elementary to middle school. In contrast, students in the PV group spent less time using computers on weekdays. It is possible that those with reduced vision limited their digital device use time. An increased time spent using digital devices decreases outdoor activities and increases the risk of myopia [23]. Consequently, it is necessary for families and schools to work together and educate students on the appropriate time and use of digital devices, starting when their vision is still good. The next environmental factor is sleep. The PV group felt that they did not get enough sleep when they were in middle school. Decreased sleep duration has been highlighted as a risk factor for myopia [24,25], and poor sleep quality is associated with faster eye axis length growth [26]. Consequently, it is possible that the students in this study might not have had enough quantity and quality of sleep and eye rest as they advanced through the school year, which may have contributed to visual acuity loss. Students in the PV group were able to identify their own health problems of sleep insufficiency.

The students in this study received instructions regarding vision loss prevention in the fourth grade. They were continuously aware of the content of the instruction from fourth grade to junior high school. The PV group students were less aware of taking breaks while studying or watching TV, suggesting that the PV group did not take breaks during near vision work. Students and families should practice setting alarms together and take regular breaks during near-sighted work.

Limitations of the study

This study has some limitations. This study used the secondary survey results conducted by the school; therefore, the content of the survey was limited. The small sample size of students from a single elementary and junior high school in Japan introduces selection bias, limiting the generalizability of the results. Therefore, further research on a broader national scale is required. The lifestyle survey was self-reported based on the students' perceptions. Therefore, there is a possibility that it does not correspond to the actual behavior of the student and may introduce recall bias. Verification through behavioral observations and the use of wearable devices is required.

## Conclusions

The study revealed several key findings, suggesting the need to teach students to prevent poor vision starting at school age while their eyesight is still good. Students with poor vision in junior high school often had poor vision as early as fourth grade. As the children advanced from middle to elementary school, the time spent using digital devices increased, whereas the time and frequency of indoor and outdoor activities reduced, and more students had difficulty sleeping. The PV group spent more time studying on weekends and less time on the computer during the week, reported insufficient sleep time, and neglected taking breaks while studying or watching TV. As students grow, they tend to spend more time studying and change their living environment to one that demands overuse of their eyes, increasing the strain on them. Students should always be made aware of the prevention of vision loss. Awareness of poor vision prevention did not change from fourth grade to middle school. It is necessary to focus on the visual acuity of middle school students and reinforce behaviors that prevent poor vision.
